# Medical Opinion and Movement

**Published:** 1910-03-05

**Authors:** 


### Medical Opinion and Movement.

### A New Disinfectant for the Hands.

Drs. Zabloudovsky and Tatarinov, of Moscow, claim to have discovered a new method of disinfecting the hands of the surgeon, which, according to their bacteriological experiments, yields most satisfactory results. It consists in rubbing the hands with sterilised gauze soaked in a 5 per cent. solution of tannin in 95 per cent. alcohol. According to the authors it matters not whether the hands be previously washed or not, wet or dry. Cultivations taken from hands treated in this way remain sterile, and the disinfection remains for some time, and is not altered by contact with liquids nor by movements or friction. After application of this solution to the hands they become smooth and soft, as if they had been polished. They have only carried out 32 operations with the hands sterilised in this manner, but in each case the result was completely satisfactory. Bacteriological tests made during operation were always negative, and even 40 and 50 minutes after operation. The operations included radical cures for hernia, thyroidectomies, and resections of joints.

### Tuberculous Infection.

At a meeting of the *Société Médicale des Hôpitaux* Dr. J. Courmont, of Lyons, gave some interesting results from experiments on animals with a view to determine the possible and probable channels of tuberculous infection. These experiments on calves, guinea pigs, and rabbits demonstrate the ease with which the bacillus of Koch is able to traverse the skin and mucous membranes, even of healthy appearance. He is of opinion that pulmonary tuberculosis is rarely primary. When the infection is directly alveolar, by inhalation, it takes the form of caseous broncho-pneumonia, but almost all cases of chronic phthisis are secondary to glandular lesions. The modes of entry of the bacilli which eventually infect the glands are multiple, and, according to this author, insufficient attention has been given to the possibilities of infection in children through skin abrasions of the face and through the mucous membranes of the mouth, nose, and eyes. The bacilli inoculated in this way penetrate to the glands without producing any local lesions. He suggests that flies, by coming in contact with tuberculous sputum, form a medium for such infections. Dr. Barbier, however, joined issue with Dr. Courmont on these conclusions. He maintained that in a considerable number of autopsies which he had carried out on tuberculous children he had almost always found the primary lesion in the lung in the form of a small caseous or fibrous focus. In further evidence of this pulmonary channel of infection he points to the frequent presence of large masses of infected mediastinal glands, from which the bacillus is able to spread in all directions through the organism.

### Eclampsia and Saline Injections.

On the theory that eclampsia is a form of auto-intoxication, subcutaneous injections of normal saline solution have been adopted as a means of treatment with a view to dilute the toxins and wash them out of the system. Dr. A. Sippel, in a contribution to the *Deutsche Medizinische Wochenschrift*, raises the question of the propriety of such treatment, at any rate in such cases where the kidneys are seriously involved. His attention was drawn to the matter by a case of eclampsia in which such injections were freely used, and appeared rather to hasten a fatal issue. He points out that with the kidneys diseased the sodium chloride injected would tend to accumulate in the tissues, instead of being eliminated by renal excretion, and so increase the œdema and cause an aggravation of the condition. In attempting, therefore, to secure a disintoxication by flushing the system with fluid and exciting a diuresis, the fluids should be introduced either into the stomach or by rectal injection, and such fluids should be as weak as possible in sodium chloride content. Even, however, if normal saline be used there is a fundamental difference between introducing the fluid into the alimentary canal and injecting it subcutaneously or intravenously. In the latter case the whole amount of salt is absorbed into the system, and must either remain in the tissues or be eliminated by the kidneys, but in the case of the alimentary canal absorption of the salt will depend upon the relative osmotic tension of the tissues. By introducing, however, fluid of low osmotic pressure into the rectum or stomach of eclamptic patients, it will tend to lower the molecular concentration of the blood, and so assist elimination of the toxins by the kidneys.

### Diphtheritic Toxins and the X-Rays.

The importance of early injections of anti-diphtheritic serum in cases of diphtheria has been constantly emphasised by authorities on the subject on account of the fact that the serum is only able to neutralise such toxins as are still circulating in the blood and have not yet been fixed in the tissues. This fixation of the toxins in the tissues takes place very rapidly, and it has been shown experimentally that in order to successfully neutralise a fatal dose of toxin injected into an animal, an interval of only a few hours must elapse before the injection of the antitoxin. To find a means of neutralising the toxins already fixed in the tisues in cases of diphtheria and of other infections still remains a problem for solution. Dr. Gerhartz has found that the *x*-rays exercise a destructive or neutralising effect upon the toxins of diphtheria. An account of his work appears in the *Berliner Klinische Wochenschrift*. He finds that a short exposure of a fatal dose of diphtheria toxin to the action of the *x*-rays is sufficient to diminish its virulence so that when injected into a rabbit the death of the animal is considerably retarded. A rabbit injected with six times the strength of a fatal dose of toxin previously exposed to the $x$-rays survived three and a-half days longer than a control rabbit that received five times the strength of a fatal dose not so treated. Dr. Gerhartz has also tested the effect of the $x$-rays on the animal itself after injection with toxin. In these cases also the animals exposed to the $x$-rays after injection lived longer than those receiving the same dose of toxin, but not so exposed. The author concludes, therefore, that the $x$-rays have a neutralising effect upon the toxins, not only *in vitro*, but also upon the toxins circulating in the blood and those fixed in the tissues. This latter point, however, would hardly appear to be proved by his experiments, as such a delay in the fatal issue might reasonably be expected if the $x$-rays only neutralised the circulating toxins, just as in the case of antitoxin injections.

### Cancer of the Sigmoid Colon.

It would seem that in regard to the extirpation of malignant growths of the upper rectum and sigmoid colon surgical opinion in Britain stands distinctly ahead of that abroad. In recent textbooks of surgery here the advantages are fully recognised of the combined abdominal and perineal removal of the growth, the bowel for some distance on each side of it, and the adjacent lymphatic tract, with the entire pelvic mesocolon in one piece—an operation which has been very prominently advocated and practised by Mr. W. E. Miles. Proctologists in this country are accepting this technique in one or other of its modifications very widely, and even surgeons, whose experience does not include much rectal work, have mostly adopted it. It is astonishing to notice, therefore, that abroad any surgeon who has done one or two of these operations immediately rushes into print with the fervour and the indefinable air of self-satisfaction of the pioneer. A month or two ago a French surgeon published his impressions of the operation after performing it twice. A modification has now just been published by an American surgeon on the strength of only one case, the essential features being the preservation of the natural outlet of the bowel by uniting its severed ends with a formidable instrument planned somewhat on the lines of Murphy's button. The one advantage of this plan is the avoidance of a colostomy; in every other respect the description of the operation leaves the impression that it is decidedly a retrograde step; most important of all is the omission to secure the entire removal of the pelvic mesocolon, in which recurrence is commonest.

### Ileus after Fractured Ribs.

Two cases of paralytic ileus of the intestine following fractures of ribs in elderly men are published by Mr. J. E. Adams, in the *Annals of Surgery*. In both cases the patients rapidly became extremely ill with symptoms suggesting intestinal obstruction, such as great distension, bilious and then fæcal vomit, and so on. In the first case the abdomen was closed after exploration had revealed the true condition, and by the energetic employment of stimulant clysters the bowels were got to act. Notwithstanding this, the patient died forty-eight hours after operation. In the second case the general condition was so bad that a general anæsthetic was impossible, and the surgeon first punctured the gut through the abdominal wall with a needle. This resulted in the escape of much gas and some fæces; the bowels then acted twice. But symptoms recurred, and sixteen hours later the man was as bad as ever. This time an incision was made under local anæsthesia, and a coil of small intestine was rapidly sutured to the wound and a rubber catheter tied into it. This time success was attained; the man rapidly recovered, and his fæcal fistula closed completely in about eight weeks. The author ascribes the ileus to splanchnic irritation by the broken ribs; the great splanchnic arises from the sixth to tenth dorsal ganglia, and the ribs affected were the eighth and ninth in one case, and the eighth to eleventh in the other. He concludes that surgeons should be less hesitant about ileostomy in these paralytic cases, done as in his successful case under local anæsthesia, and with the greatest possible rapidity.

### A Note on Sigmoidoscopy.

In a contribution to the *Northumberland and Durham Medical Journal*, Mr. Hamilton Drummond outlines the preliminary treatment which experience has shown to be essential for successful use of the sigmoidoscope. Two days before the examination, preferably at bed time, a full dose of castor oil or of blue pill with colocynth and hyoscyamus is given; this is followed in the morning by a saline aperient, and then by a two-pint enema. On the morning of the day of examination a succession of simple enemata is given until the fluid returns clear. The examination takes place about two hours after this happens. During this period of preparation the patient is kept on a low diet. In cases of exceptional difficulty, as in colitis, a longer time is occupied in preparation. In these cases no aperient is given in the three days preceding sigmoidoscopy, but many simple enemata are used, and an astringent mixture is also given. The author describes the technique of introduction of the instrument, and explains how to avoid rupturing the gut. This accident, there is little doubt, is a great deal commoner than most surgeons suppose. Cases are not often published, for obvious reasons; but there were several in London alone last year, and nearly every surgeon who uses this instrument seems to have experienced this disaster once at least. It would be interesting if some proctologist would endeavour, by confidential inquiry, to find out what the collective experience of surgeons really is on this point. The result would probably surprise the profession, which is somewhat too apt to accept the assurances of expert rectal specialists that the use of the instrument in careful hands is almost devoid of risk.

### Trephining the Globe for Glaucoma.

In the December and February numbers of the *Ophthalmoscope* are two descriptions by Major R. H. Elliot and Dr. Freeland Fergus respectively of this new operation for the relief of glaucoma. Curiously enough, the idea is a very old one, for it was first devised and employed 34 years ago by Argyll Robertson, who abandoned it after unsatisfactory results. As now revived the operation differs in important particulars from Robertson's original method. Dr. Fergus, after dissecting a large conjunctival flap up to the sclero-corneal margin, removes with a Bowman's trephine a piece of sclera as close to the cornea as possible. The point of an iris repositor is next passed from the scleral opening into the anterior chamber. The conjunctival flap is then replaced and sewn into position. This operation was conceived by the author from a suggestion by Lagrange, and it has affinities also to Heine's cyclodialysis. Major Elliot's technique is rather different. He reflects a flap of conjunctiva, applies the crown of a two-millimetre trephine as close to the limbus as possible, and aims at allowing the instrument to cut its own way into the anterior chamber. The disc of sclera may be left in place, or removed, at pleasure. The aim of this manœuvre is to establish a permanent filtering cicatrix between the anterior chamber and the subconjunctival space. Major Elliot claims that a tyro can accomplish thus all that Herbert and Lagrange aim at without mastering the difficult detail of their operations. Extended experience among ophthalmologists should soon determine which, if either, of these operations is most calculated to achieve the ends in view, which are not merely the preservation of sight in those recently affected, but also the relief of the distressing pain which eyes already blinded beyond repair frequently give rise to. Further, it is interesting to note that opinions given at a recent meeting of the Ophthalmological Society are decidedly against Lagrange's method.

### Disease of the Thyroid and Lactation.

It is well-known that congenital myxœdema only manifests itself when the child is weaned, a fact which leads to the belief that maternal milk has some therapeutic influence in such cases. Professor Spolverini has had the opportunity of observing in infants disturbances of hyper- and hypothyroidism corresponding to the pathological condition of the thyroid gland of the mother or nurse. He reports his observations in the *Revue d'Hygiène et de Médecine*. The first two cases concern two infants, nursed successively by the same foster-mother, who both showed symptoms of myxœdema, which rapidly disappeared under the influence of thyroid treatment. In consequence of the phenomena exhibited by the second child the nurse was examined, and found to have a goître, with some signs of hyper-thyroidism. In the third case the nurse of a healthy infant was changed. Three weeks later the latter showed a hard œdema of the limbs, with swelling of the tongue. The nurse was examined and found to have a goître. Her charge was removed from her care and its symptoms rapidly disappeared. In the fourth and fifth cases the children were nursed by their own mothers, who both had thyroid tumours. After a few days in each case the infants showed symptoms of hypothyroidism. Rapid improvement followed when the mothers were put on thyroid medication. The last observation concerns a woman with Basedow's disease who had three children. The one she was nursing at the time exhibited signs of hyperthyroidism and died. The others, who had been suckled by wet-nurses, had no morbid symptoms.

### Genital Herpes.

Dr. Emery discusses the diagnosis and treatment of genital herpes in a recent number of *La Clinique*. The affection is acute, runs a definite course, and then rapidly disappears. It is characterised by an eruption, usually vesicular, the vesicles occurring in clumps and rapidly becoming ulcerated, the ulcers being rounded in shape. In the male both the skin and the mucous surfaces are usually attacked, but in the female the vesicles are confined to the mucous surfaces. Despite its well-marked characters, the affection is often confounded both with soft and Hunterian chancres. Microscopical investigations for the bacillus of Ducrey and the treponema pallidum respectively will alone provide the key to the diagnosis in such cases. Other affections for which genital herpes may be mistaken are traumatic ulceration, eczema of the prepuce and glans penis, and circumscribed balanitis. Local treatment, which is often superfluous, must in all cases be of the simplest kind, such as emollient lotions, ointments, inert powders. In cases where there is much inflammation fomentations may be used. Herpes of the mucous surfaces should be treated with antiseptic lotions and covered with protective dressings, but no caustic should be applied. The nervous phenomena associated with the affection should receive appropriate treatment, and attention should be paid to the general health and the ordinary hygienic rules rigidly adhered to.

### The Soap Enema.

It is well known that cutaneous eruptions sometimes follow the use of a soap enema. F. J. Shepherd, in the *Journal of Cutaneous Diseases*, reports the results of his inquiries into the cause of these phenomena. His investigations led him to trace the cause to the fact that common yellow soap is frequently used in enemata given prior to operation. On the other hand, he found that no evil results followed when an enema of Castile soap was given to patients who had suffered from eruptions after the use of a common soap enema. On analysing common yellow soap the writer discovered that a considerable quantity of resin was present, and attributes the cause of many rashes seen after abdominal operations to the presence of this substance in the soap enema given at the time of preparation.

